# Reducing Delusional Conviction through a Cognitive-Based Group Training Game: A Multicentre Randomized Controlled Trial

**DOI:** 10.3389/fpsyt.2015.00066

**Published:** 2015-04-28

**Authors:** Yasser Khazaal, Anne Chatton, Karen Dieben, Philippe Huguelet, Maria Boucherie, Gregoire Monney, Laurent Lecardeur, Virginie Salamin, Fethi Bretel, Silke Azoulay, Elodie Pesenti, Raoul Krychowski, Andreia Costa Prata, Javier Bartolomei, Perrine Brazo, Alexei Traian, Thomas Charpeaud, Elodie Murys, Florent Poupart, Serge Rouvière, Daniele Zullino, Alberto Parabiaghi, Mohamed Saoud, Jérôme Favrod

**Affiliations:** ^1^Geneva University Hospitals, Geneva University, Geneva, Switzerland; ^2^Service de Psychiatrie, CHU de Caen, Université de Caen Basse-Normandie, UMR6301 ISTCT, ISTS Team, Caen, France; ^3^Fribourg Network for Mental Health, Fribourg, Switzerland; ^4^Service de Psychiatrie Ambulatoire et de Réhabilitation du Pôle Rouen Rive Droite, Centre Hospitalier du Rouvray, Centre de Jour Saint-Gervais, Rouen, France; ^5^Centre Psychosocial, Biel, Switzerland; ^6^MIDI Foyer SISP EMS, Yverdon Les Bains, Switzerland; ^7^La Borde, Foyer SISP EMS, Lausanne, Switzerland; ^8^Centre Medico-Psychologique B, Centre Hospitalier Universitaire, Clermont-Ferrand, France; ^9^Service de Psychiatrie, Centre Hospitalier de Vichy, Vichy, France; ^10^Unité Mobile de Psychiatrie, Centre Hospitalier Princess Grace, Monaco, Monaco; ^11^Léon-Jean Grégory Hospital, Thuir, France; ^12^CRESOP, Centre Hospitalier le Vinatier, Bron, France; ^13^Laboratory of Epidemiology and Social Psychiatry, IRCCS ‘Mario Negri’ Institute for Pharmacological Research, Milan, Italy; ^14^EA 4615, Université de Lyon, Lyon, France; ^15^Université Lyon 1, Lyon, France; ^16^School of Nursing Sciences La Source, University of Applied Sciences and Arts of Western Switzerland, Lausanne, Switzerland

**Keywords:** psychotic disorders, schizophrenia, cognitive therapy, game, hypothetical reasoning, randomized controlled study, psychotherapy

## Abstract

**Objective:**

“Michael’s game” (MG) is a card game targeting the ability to generate alternative hypotheses to explain a given experience. The main objective was to evaluate the effect of MG on delusional conviction as measured by the primary study outcome: the change in scores on the conviction subscale of the Peters delusions inventory (PDI-21). Other variables of interest were the change in scores on the distress and preoccupation subscales of the PDI-21, the brief psychiatric rating scale, the Beck cognitive insight scale, and belief flexibility assessed with the Maudsley assessment of delusions schedule (MADS).

**Methods:**

We performed a parallel, assessor-blinded, randomized controlled superiority trial comparing treatment as usual plus participation in MG with treatment as usual plus being on a waiting list (TAU) in a sample of adult outpatients with psychotic disorders and persistent positive psychotic symptoms at inclusion.

**Results:**

The 172 participants were randomized, with 86 included in each study arm. Assessments were performed at inclusion (T1: baseline), at 3 months (T2: post-treatment), and at 6 months after the second assessment (T3: follow-up). At T2, a positive treatment effect was observed on the primary outcome, the PDI-21 conviction subscale (*p* = 0.005). At T3, a sustained effect was observed for the conviction subscale (*p* = 0.002). Further effects were also observed at T3 on the PDI-21 distress (*p* = 0.002) and preoccupation subscales (*p* = 0.001), as well as on one of the MADS measures of belief flexibility (“anything against the belief”) (*p* = 0.001).

**Conclusion:**

The study demonstrated some significant beneficial effect of MG.

## Introduction

A considerable proportion of patients with psychotic disorders do not respond fully to antipsychotic agents (1). As an adjunct treatment, however, cognitive behavioral therapy (CBT) has, to some extent, a favorable effect on psychotic symptoms (2–7), awareness of illness, distress, preoccupation, conviction, and behavioral consequences of delusional beliefs (8–11). A core component of CBT is based on hypothetical reasoning, which consists of the search for alternative explanations for a given experience (12). Delusions are associated with low belief flexibility, with most patients unable to spontaneously find alternative explanations for their beliefs (13). Promoting an alternative hypothesis may reduce their degree of conviction, preoccupation, and distress associated with delusions (14, 15).

Despite an increasing need for trained professionals who are able to deliver CBT for psychotic symptoms in naturalistic settings, studies have emphasized that training opportunities are lacking and that the numbers of qualified therapists are poor (16–18). Moreover, most studies in the field have been performed in CBT specialized settings with highly selected patients (19). A number of preliminary studies have shown, however, that new tools that integrate CBT techniques in game format (20, 21) and some forms of computer-assisted therapy (22) represent promising treatment for patients with psychotic disorders.

“Michael’s game” (MG) (Table 1) is a hypothetical reasoning training module that promotes the dissemination of CBT (specifically, reasoning training) in natural clinical settings. It is based on CBT of psychotic symptoms (5, 23, 24), as described elsewhere (20, 21). The aim of the game is to train people to find alternative hypotheses for a given situation.

**Table 1:** **“Michael’s game”**.

**Examples of objectives on the cards**
Describe a situation before interpretation
Devise the interpretation of a situation as a hypothesis
Search for different interpretations of the same situation
Identify the cognitive and behavioral consequences of the different hypotheses
Search for a link between the interpretation given for a situation and a personal real-life experience
Put the hypotheses in hierarchical order in terms of their probability
Search for arguments for or against a hypothesis
Think of a way of testing a given hypothesis in reality
**Examples of cards**
*A non-psychotic and non-emotional situation card*
Michael sets two bags of different sizes on each side of a scale
The big bag has the same weight as the small bag
Michael is surprised since the two bags are supposed to be filled with cotton
He thinks that the small bag contains a stone
*A psychotic card*
Michael is watching his favorite show on television
When the show host appears, Michael is so pleased that he bursts out laughing
The show host and another participant in the show start laughing at the same time
Michael tells himself: “My joy is contagious”

*The game was conceived by two of the authors, Yasser Khazaal and Jerome Favrod. It has been translated into English, French, Spanish, German, and Italian. The game includes non-psychotic, non-emotional cards (1–11); emotional, non-psychotic cards (12–32); and psychotic cards (33 to the end)*.

Two preliminary studies (20, 21) support the possible impact of the game on psychotic symptoms.

Those earlier studies had, however, a number of limitations such as lack of control group, lack of blind assessment, the non-controlled character of the pharmacological treatments, as well as the lack of follow-up measures.

Furthermore, the consistency of the intervention was not controlled by audio recording of the sessions. The study at hand aimed to overcome the weakness of the previous works with more rigorous methods such as a randomized controlled design, blind assessments, sessions recording, and a follow-up assessment.

In the present randomized controlled trial, we hypothesized a more important impact of “treatment as usual + Michael’s game” (MG) than of “treatment as usual + waiting list” (TAU) on a measure of psychotic symptoms related to conviction (primary outcome) and possibly on measures of distress, preoccupation, symptom intensity, belief flexibility, cognitive insight, awareness of illness, and actions on beliefs (secondary outcomes).

The aim of the trial is to assess the short-term effect (post-intervention) of MG in comparison to TAU on the primary outcome and on further secondary outcomes. The sustained or long-term effect of the intervention in comparison to TAU was also evaluated 6 months after the post-intervention assessment of the primary and secondary outcomes.

## Materials and Methods

Participants were outpatients recruited in psychiatric rehabilitation units and outpatient clinics in Switzerland, France, Monaco, and Italy. Potential participants were identified through systematic screening of medical records. Written informed consent was obtained from all participants. Institutional review boards and the ethical committees in Switzerland, France, and Italy approved the study protocol. The protocol was also made available to all study investigators and was registered (International Standard Randomized Controlled Trial Number Register: ISRCTN37178153)^1^. The study was carried out from October 2008 to September 2011. The study’s duration was longer than initially expected because of a delay in the recruitment process. The study had, however, slightly higher recruitment of participants than first planned (172 rather than 166) and a higher retention rate than originally expected (124 patients assessed at the end point rather than the 94 projected). There were no other deviations from the original study protocol.

### Randomization

Eligible patients were randomized to either TAU or to MG after providing informed consent. The randomization scheme was generated by using the website randomisation.com^2^ and kept independently by a statistician. The allocation ratio used was one to one. A permuted-block randomization procedure was used with fixed block sizes of four patients, ensuring that the number of subjects in the different groups closely balanced at all times. The study investigators, who were in charge of enrollment, were blinded to the randomization sequence in order to prevent them from predicting patient allocation, thus reducing selection bias. There was no randomization by center.

### Assessment procedures

Patients were assessed at baseline (T1); at 3 months, after the end of the MG sessions (T2); and 6 months after the second assessment (T3). Psychologists or psychiatrists made the assessments independently from the therapists, and the game leaders and were not informed of the treatment received by the participants (blind evaluation). The patients were given 40 Swiss francs (approximately 35 Euros or 40 US Dollars) as compensation after each evaluation.

### Measures

- Mini-international neuropsychiatric interview (MINI) (25) for psychiatric diagnosis according to the *DSM-IV*.
- Peters delusions inventory (PDI-21) (26, 27). Multidimensionality of delusions is approached in the PDI-21 by measuring distress, preoccupation, and conviction related to each of 21 stated beliefs on a 5-point Likert scale. Patients with psychotic disorders differ from controls by having higher ratings on distress, preoccupation, and conviction scales (26). The scales were proposed as a possible measure of change during CBT (26). Results of preliminary studies (20, 21) showed a treatment × time effect on the scores of the PDI-21.
- Brief psychiatry rating scale (BPRS) (28). The following scores were considered: affect, positive symptoms, negative symptoms, resistance, activation (29), and total score.
- Beck cognitive insight scale (BCIS) (30, 31). The BCIS is composed of two subscales: self-reflectiveness related to the ability to consider alternate explanations and openness to feedback, and self-certainty related to the degree of certainty and confidence related to beliefs. A BCIS composite index is obtained by subtracting the self-certainty from the self-reflectiveness score. It was hypothesized (32) that low self-reflectiveness and high self-certainty (low composite index) may constitute a reasoning style that would maintain delusional beliefs.
- Global assessment of functioning (GAF) scale (DSM-IV).
- Social and occupational functioning assessment scale (SOFAS).
- Maudsley assessment of delusions schedule (MADS) (33). MADS is a standardized interview designed to evaluate the phenomena related to the principal abnormal belief of a patient. In practice, the MADS improves the assessment of the possible impact of the game on specific and individualized aspects of the main delusional idea of each included patient and on measures of belief flexibility specifically linked with the main delusional belief. The following MADS assessments were included in the study:
- Belief flexibility. As suggested elsewhere (15), the participants were asked whether or not it was possible that they may be mistaken about their main belief (possibility of being mistaken). They were also presented with a hypothetical scenario, which, if true, would contradict the delusion, and asked how this would change their belief (change conviction). The participants were also asked whether or not anything has happened that goes against the belief and “What would have to happen to make you think that you might be wrong about this belief?” This assessment could be considered as a form of “ability to plan a behavioral experiment.”
- Awareness of illness.

### Primary outcome

The primary outcome was the change after treatment in the conviction score of the PDI-21.

### Secondary outcomes

The 16 secondary outcomes included the change in scores on the other two subscales of the PDI-21 (distress and preoccupation), the five subscales of the BPRS, the two subscales of the BCIS, the GAF, the SOFAS, and the five items of the MADS.

### Sample size and power

An *a priori* sample size was estimated from a pilot study (21), using the conviction subscale of the PDI-21 in the calculation. By using the Diggle formula for the sample size calculation in longitudinal data, we found that a sample size of at least 47 persons per group was needed at each study evaluation (error rate of 0.05; statistical power of 0.8).

### Inclusion criteria

- Psychotic disorder according to the DSM-IV (diagnostic based on chart review and MINI)
- Outpatient setting
- 18–65 years old
- Persistent positive psychotic symptoms at inclusion: BPRS score of ≥3 on at least two items of the positive symptoms BPRS subscale

### Exclusion criteria

- Organic brain disease
- Mental retardation
- BPRS conceptual disorganization score >5
- Prior participation in MG
- Cognitive therapy of psychotic symptoms at inclusion

### Interventions

#### Michael’s Game

Michael’s game is a collaborative group game consisting of 80 cards (Table 1). Each card corresponds to a situation and to objectives that target the ability to reason with hypotheses (see Table 1 for an example of a card). Training group leaders (two per session) direct the game during weekly sessions lasting for about 1 h. Because MG is an 80-card game, the mean number of sessions needed to end the game is not *a priori* fixed. The mean number of sessions needed to end the game was 12.1 (SD = 3.41). The mean participation rate of the participants (number of sessions completed by a participant × 100/total number of sessions needed to complete the program in the same group) was 91.2% (SD = 14.1). These figures are similar to those described in the preliminary reports related to the game (20). Failure to attend more than three sessions was considered to be MG treatment discontinuation. Participants (in groups of four to eight patients) are led through specific questions to find multiple answers (hypotheses) to the questions and conclusions that Michael draws from the situations that he is confronted with. In other words, participants have to help Michael to find alternatives to the conclusions that he draws from situations described on each card. MG was conceived as a collaborative group card game in order to allow patients to become partners of a fictive character (Michael) and together to interact with cards containing impersonal information that may reflect their own concerns. Participants play together on objectives such as the following: describe the situation reported on a given card, identify Michael’s hypothesis, search for different hypotheses that may explain the same situation, report the possible cognitive and behavioral consequences of the different hypotheses, give arguments for or against a given hypothesis, and imagine how to test a given hypothesis in reality. Game directors were psychiatric care workers (nurses: 14, psychologists: 12, psychiatrists: 6) who were specifically trained to deliver MG according to the training model described below.

#### Treatment as Usual Plus Waiting List (TAU; Control Condition)

Treatment as usual comprises case management, psychosocial interventions, antipsychotic medication, and outpatient and community follow-up. The patients in the TAU condition had to wait until the end of follow-up before participating in a MG.

The collaborative centers had access to similar pharmacological treatment, psychosocial structures, and level of medical training. Medication was monitored during the study. Chlorpromazine equivalences were calculated for antipsychotic medications according to Woods (34). Participants from both groups received treatment as usual throughout the entire study period.

### Quality assurance

Group directors at each of the 16 collaborative centers (nurses, psychologists, psychiatrists) were trained by the same method: a standardized 1 h presentation and a 1 h training session for the game. Training was directed by one of the game’s authors or by one person authorized by the authors for his or her educational qualities and experience in directing the game. Supervision was also offered to game directors by the first author upon request. Sessions were audiotaped (not available for all centers) to check their fidelity to the intervention and were assessed with expected therapist strategies extracted from the collaboration, interpersonal effectiveness, understanding, and guided discovery items of the Haddock scale (35). Twenty-five randomly selected sessions were assessed. The percentage of items rated as being compliant with the desirable strategies ranged from 72 to 100%.

### Statistical analyses

At the patient level, baseline differences were checked between the two study groups, as well as differences between completers and non-completers using *t*-tests, chi-square tests, or Fisher’s exact tests where applicable.

The direct effect, or the short-term effect, of treatment on the primary and on the secondary continuous outcomes was evaluated through analyses of covariance (ANCOVAs) by considering outcome values after the intervention at T2 and adjusting for the respective baseline outcomes. The treatment group served as the between-subject factor. These analyses were also controlled for center to correct for a possible cluster effect and for medication. Similarly, the long-term effect at T3 of the intervention was measured by ANCOVAS, using outcome values at T3 and adjusting for the respective baseline outcomes. Finally, categorical MADS variables were analyzed at T2 (short-term effect) and T3 (long-term effect) through logistic regression analyses, with treatment group as the between-subject factor, and were also adjusted for baseline outcomes and controlled for center and medication. The necessary application conditions of all these models were checked and met.

#### Adjusting for Multiple Testing

The trial was *a priori* powered for multiple time points at a predefined 0.05 significance level, using PDI-21 conviction as the primary outcome. Therefore, this level of significance was kept when the primary outcome results were interpreted. However, to minimize the chance of spurious results pertaining to the secondary outcomes, we adjusted them for multiple testing by Bonferroni’s correction (α ≤ 0.05/16 = 0.0031). All the statistical analyses were performed with the Statistical Package for the Social Sciences (SPSS version 18.0, IBM, Chicago, IL, USA).

#### Treatment of Missing Data

Analyses were done on an intention-to-treat basis, whereby all randomized subjects were included. Categorical missing data were imputed using logistic regressions. Quantitative missing data were imputed by the expectation–maximization algorithm, a statistical simulation technique that estimates the averages, the matrix of variance and covariance, and the matrix of correlations. After convergence, missing data are replaced by the estimation obtained and the completed data are then analyzed by the usual methods.

## Results

**Figure 1:**
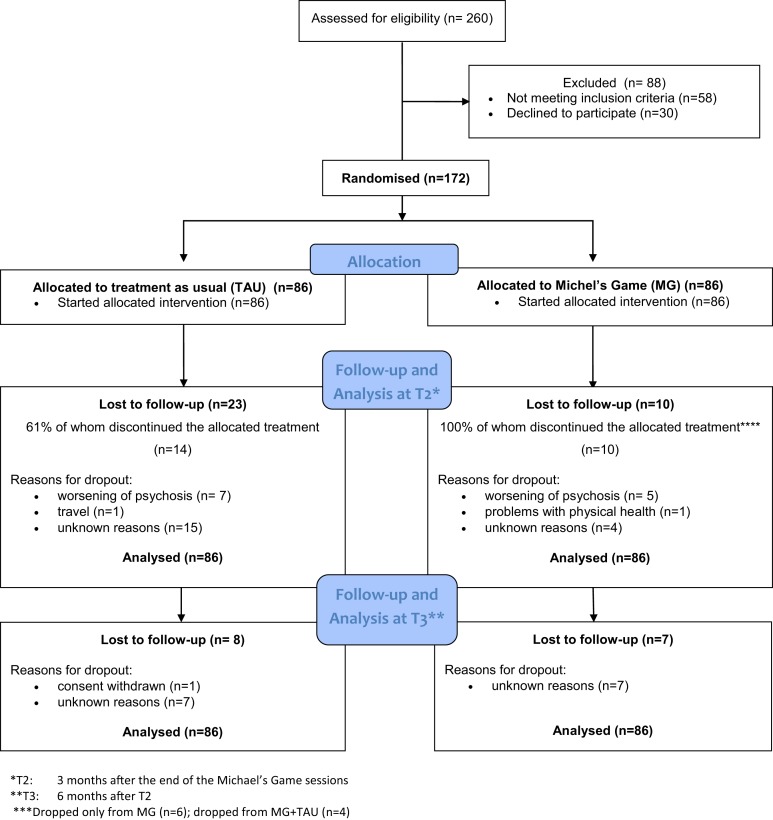
**CONSORT flow diagram**.

After screening and informed consent, 172 patients were included and randomized for study participation (Figure 1).

### Baseline characteristics of subjects

The mean age of the sample was 37.1 years (SD = 10.4). A high school diploma or a university degree had been attained by 27.1% of participants. Most participants had a diagnosis of schizophrenia (81.4%), were single (78.5%), lived in a private residence (59.3%), and had a disability pension (73.1%). Concurrent psychiatric disorders at inclusion were common [i.e., substance use (15.6%); anxiety disorders (27.2%)].

At baseline, the PDI-21 distress score was higher for the MG group than for the TAU group at a trend level (*p* = 0.05). There were no differences in the other main clinical measures (Table 2).

**Table 2:** **Baseline sociodemographic and clinical characteristics of the participants in the Michael’s game (MG) and the treatment as usual (TAU) groups**.

Baseline variables	TAU	MG	*p*-Value
	(*n* = 86)	(*n* = 86)	
		
	Mean (SD) or %	Mean (SD) or %	
Age	37.1 (10.8)	37.0 (10.1)	0.9
Gender: male	67.4	57.0	0.2
Marital status: single	77.9	79.1	0.9
Recruiting regional centers			0.9
Switzerland	47.7	51.2	
France/Monaco	39.5	37.2	
Italy	12.8	11.6	
Highest educational degree obtained			0.4
Primary/grammar school	43.5	50.6	
Apprenticeship/professional school	24.7	27.1	
High school/university	31.8	22.4	
Diagnosis			0.2
Schizophrenia	77.9	84.9	
Other psychotic disorders	22.1	15.1	
PDI			
Distress	21.0 (17.3)	25.3 (16.2)	0.05
Preoccupation	21.1 (15.8)	23.9 (14.9)	0.1
Conviction	27.0 (18.3)	29.8 (18.4)	0.1
BCIS			
Self-reflectiveness	14.4 (5.0)	15.1 (4.6)	0.3
Self-certainty	8.4 (3.1)	8.9 (4.1)	0.2
Composite index	5.9 (6.3)	6.3 (6.5)	1.0
MADS: anything against the belief			
Yes answers (%)	38.4	31.4	0.1
MADS: possibility of being mistaken			
Yes answers (%)	57.0	52.3	0.7
MADS: response to hypothetical contradiction (%)			
Dismisses belief	25.6	12.8	0.2
Changes conviction	15.1	15.1	
Accommodates	29.1	30.2	
Ignores or rejects	30.2	41.9	
MADS: ability to plan a behavioral experiment (%)			
Able to outline evidence and this outcome logically possible	36.0	29.1	0.4
Able to outline evidence but this outcome logically impossible	12.8	9.3	
Unable to outline evidence which would contradict his belief	51.2	61.6	
MADS: awareness of illness (%)			
Accept that has a mental illness or nervous problem which includes delusional belief	66.3	52.3	0.2
Accept that has a mental illness or nervous problem but does not include delusional belief	19.8	30.2	
Not ill	14.0	17.4	
BPRS			
Affect	10.5 (4.2)	11.4 (4.3)	0.2
Negative symptoms	8.6 (3.9)	8.7 (3.9)	0.9
Positive symptoms	11.1 (4.4)	11.4 (3.3)	0.7
Resistance	7.2 (2.9)	7.6 (2.6)	0.2
Activation	5.8 (2.5)	5.4 (2.6)	0.4
BPRS total score	42.8 (11.3)	44.5 (10.2)	0.3
GAF	43.6 (13.2)	43.0 (9.4)	0.8
SOFAS	43.7 (11.6)	43.6 (9.8)	1.0
Chlorpromazine equivalent (mg/day)	269.4 (222.1)	305.9 (297.9)	0.4

*Summary statistics report means and SDs or percentages*.

*TAU, treatment as usual plus waiting list; MG, treatment as usual plus Michael’s game; PDI, Peters delusions inventory; BCIS, Beck cognitive insight scale; MADS, Maudsley assessment of delusions schedule; BPRS, brief psychiatry rating scale; GAF, global assessment of functioning; SOFAS, social and occupational functioning assessment scale*.

### Dropouts

At T2, dropout was associated with treatment allocation (*p* = 0.01). Twenty-three of the TAU group patients and 10 of the MG group patients discontinued the study. At T2, dropout was also associated with younger age (*p* = 0.02). Furthermore, dropouts were more common among patients who dismiss the belief in response to hypothetical contradiction (*p* = 0.04) and those more able to plan a behavioral experiment (*p* = 0.04). There was no other statistically significant difference on the other sociodemographic and clinical characteristics. Dropout at T3 was associated with a younger age (*p* = 0.05) and recruiting centers, where Switzerland had a statistically significant high rate of dropouts during this period compared with the other centers (*p* = 0.01). No other statistically significant difference was observed.

### Outcomes

**Figure 2:**
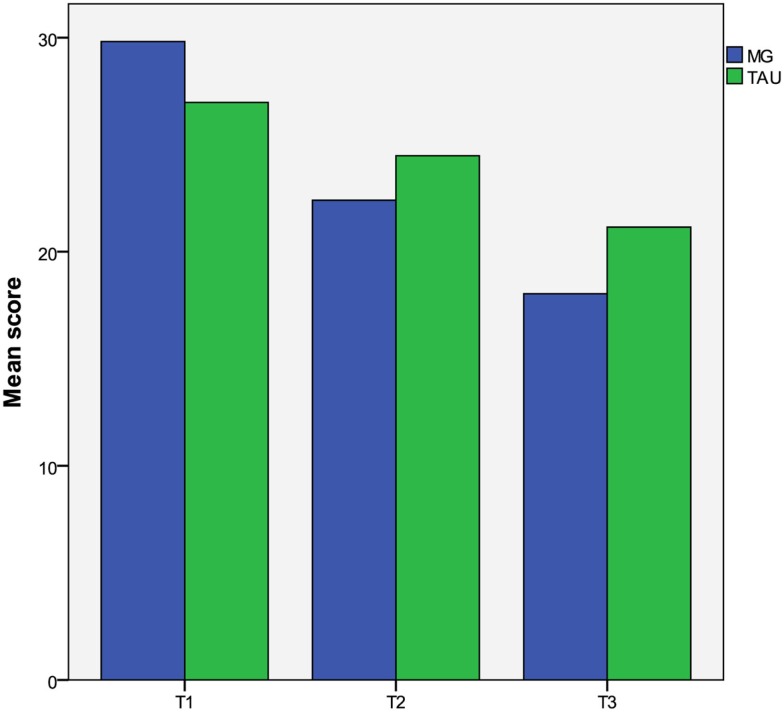
**Evolution of PDI conviction raw scores by treatment group**.

Table 3 presents patient outcomes at baseline, 3 months, and 9 months for the MG and TAU groups. Figure 2 depicts the evolution of the PDI conviction raw scores.

**Table 3:** **Evolution of primary and secondary outcome variables by treatment group**.

Outcome variable	T1		T2		T3		*p*-Value^a^^,^^b^	*p*-Value^b^^,^^c^
	TAU	MG	TAU	MG	TAU	MG	
PDI scores								
Distress	21.0 (17.3)	25.3 (16.2)	19.7 (15.3)	20.2 (14.1)	16.9 (17.0)	15.1 (12.3)	n.s.^d^	**0.002**
Preoccupation	21.1 (15.8)	23.9 (14.9)	19.6 (13.9)	18.5 (13.2)	16.9 (14.5)	14.3 (12.7)	n.s.	**0.001**
Conviction	27.0 (18.3)	29.8 (18.4)	24.5 (17.2)	22.4 (15.7)	21.2 (16.3)	18.0 (14.3)	**0.005**	**0.002**
BCIS score								
Self-reflectiveness	14.4 (5.0)	15.1 (4.6)	14.4 (5.0)	16.4 (5.0)	14.3 (4.3)	15.4 (4.6)	n.s.	n.s.
Self-certainty	8.4 (3.1)	8.9 (4.1)	8.2 (3.6)	7.9 (3.8)	8.3 (3.5)	7.7 (3.0)	n.s.	n.s.
Anything against the belief								
Yes answers (%)	38.4	31.4	33.7	48.8	36.0	53.5	n.s.	**0.001**
Possibility of being mistaken							n.s.	n.s.
Yes answers (%)	57.0	52.3	54.7	61.6	51.2	69.8		
Response to hypothetical contradiction (%)							n.s.	n.s.
Dismiss belief	25.6	12.8	41.9	25.6	53.5	36.0		
Change conviction	15.1	15.1	9.3	31.4	8.1	23.3		
Accommodate	29.1	30.2	19.8	30.2	19.8	31.4		
Ignore or reject	30.2	41.9	29.1	12.8	18.6	9.3		
Ability to plan a behavioral experiment (%)							n.s.	n.s.
Able to outline evidence and this outcome logically possible	36.0	29.1	51.2	45.3	61.6	61.6		
Able to outline evidence but this outcome logically impossible	12.8	9.3	10.5	10.5	7.0	16.3		
Unable to outline evidence which would contradict his belief	51.2	61.6	38.4	44.2	31.4	22.1		
Awareness of illness (%)							n.s.	n.s.
Accept that has a mental illness or nervous problem which includes delusional belief	66.3	52.3	65.1	69.8	80.2	79.1		
Accept that has a mental illness or nervous problem but does not include delusional belief	19.8	30.2	14.0	18.6	10.5	12.8		
Not ill	14.0	17.4	20.9	11.6	9.3	8.1		
BPRS score								
Affect	10.7 (4.2)	11.4 (4.3)	10.9 (3.9)	10.5 (4.2)	11.3 (4.1)	10.8 (4.0)	n.s.	n.s.
Negative symptoms	8.4 (3.8)	8.7 (3.9)	9.1 (3.6)	8.1 (3.8)	8.5 (3.0)	8.9 (3.9)	n.s.	n.s.
Positive symptoms	11.1 (4.3)	11.4 (3.3)	10.3 (4.1)	9.8 (4.0)	10.0 (3.8)	9.8 (3.8)	n.s.	n.s.
Resistance	7.1 (2.9)	7.6 (2.6)	6.3 (2.6)	6.4 (2.6)	6.6 (2.7)	6.3 (2.6)	n.s.	n.s.
Activation	5.5 (2.4)	5.4 (2.6)	5.4 (2.5)	5.3 (2.6)	5.5 (2.4)	5.5 (2.8)	n.s.	n.s.
GAF	43.5 (13.0)	43.0 (9.4)	47.5 (11.7)	48.0 (10.5)	47.0 (11.2)	48.7 (10.2)	n.s.	n.s.
SOFAS	43.8 (11.6)	43.7 (9.8)	47.1 (11.7)	48.4 (10.9)	46.4 (10.7)	49.0 (10.0)	n.s.	n.s.

*^a^*p*-value resulting from analysis of short-term treatment effect*.

*^b^Only significant results after Bonferroni’s correction are reported*.

*^c^*p*-value resulting from analysis of long-term treatment effect*.

*^d^n.s., not significant*.

*Interval variables are reported by their mean and SD [M (SD)] and categorical variables by their percentage (%)*.

*TAU, treatment as usual plus waiting list; MG, treatment as usual plus Michael’s game; T1, baseline; T2, at 3 months post-treatment; T3, at 6 months follow-up; PDI, Peters delusions inventory; BCIS, Beck cognitive insight scale; BPRS, brief psychiatry rating scale; GAF, global assessment of functioning; SOFAS, social and occupational functioning assessment scale*.

*Bold font indicates significant *p*-value*.

#### Short-Term Treatment Effect (at T2) on the Primary Outcome

After baseline values, medication, and the cluster effect of center were controlled for, the results of the ANCOVAS showed a short-term treatment effect for conviction (*p* = 0.005). After the intervention, the estimated mean conviction was 29.0 for the TAU group, whereas it was 24.3 for the MG group, which means a short-term treatment effect of −4.7 points (95% CI [−7.9 to −1.4]).

#### Short-Term Treatment Effect (at T2) on the Secondary Outcomes

No short-term effect was observed for the PDI-21 distress subscale (*p* = 0.1), but an effect was observed for preoccupation (*p* = 0.025), with an estimated mean of 22.3 for the TAU group and 19.0 for the MG group, a treatment effect of −3.3 points (95% CI [−6.2 to −0.4]). However, after Bonferroni’s correction, this effect was no longer significant.

No short-term treatment effect was observed for either Beck self-reflectiveness (*p* = 0.08) or Beck self-certainty (*p* = 0.4). For BPRS subscores (activation, affect, positive symptoms, and resistance), no treatment effect was found (*p* = 0.6, *p* = 0.3, *p* = 0.4, and *p* = 0.4, respectively). For BPRS negative symptoms, a treatment effect was observed (*p* = 0.019) that was not sustained after Bonferroni’s correction. GAF and SOFAS did not exhibit a treatment effect (*p* = 0.1 and *p* = 0.9, respectively). With regard to the MADS items, there was an effect for “anything against the belief” in which the MG group was more likely to answer “yes” than the TAU group (OR = 3.16, 95% CI [1.30–7.70], *p* = 0.01). This effect, however, was no longer significant after Bonferroni’s correction.

#### Long-Term Treatment Effect (at T3) on the Primary Outcome

A sustained effect of the intervention was observed for conviction (*p* = 0.002). At T3, the estimated mean conviction was 24.6 for the TAU group, whereas it was 18.8 for the MG group, a long-term treatment effect of −5.7 points (95% CI [−9.3 to −2.2]).

#### Long-Term Treatment Effect (at T3) on Secondary Outcomes

A treatment effect was observed for distress, preoccupation, GAF, and SOFAS (*p* = 0.002, *p* = 0.001, *p* = 0.03, and *p* = 0.02, respectively). The mean scores for distress and preoccupation were higher in the TAU group, whereas the mean scores for GAF and SOFAS were higher in the MG group than in the TAU group. However, after Bonferroni’s correction, the significance remained only for distress and preoccupation, for which treatment effects were estimated at −5.5 points (95% CI [−9.0 to −2.0]) and −5.5 points (95% CI [−8.6 to −2.2]), respectively.

Regarding MADS variables, a sustained treatment effect was observed only for “anything against the belief”: the MG group was more likely to answer “yes” than the TAU group (OR = 4.95, 95% CI [1.85–13.27], *p* = 0.001). This effect remained significant after Bonferroni’s correction.

## Discussion

This study investigated the effects of a hypothetical reasoning training module of short duration, called MG, in a sample of 172 outpatients with psychotic symptoms in 16 centers in France, Italy, Monaco, and Switzerland. In accord with the main hypothesis, the conviction score measured with the PDI-21 was improved in the MG condition compared with the TAU condition at T2, the post-intervention assessment. After Bonferroni’s correction, no other effects on the secondary outcomes were observed at post-intervention.

Six months later, the effect observed on the conviction score of the PDI-21 was sustained. Furthermore, other effects were observed, after Bonferroni’s correction, on the distress and preoccupation subscales of the PDI-21. In addition, these results in the MG group were supplemented with an improvement in belief flexibility, as measured with the MADS item “anything against the belief.”

The results do not show specific effects on the BPRS scale. The observed effects occurred on more detailed measures such as the conviction subscale of the PDI-21 (the main outcome), the distress and preoccupation subscales of the PDI-21, and one of the MADS items. This pattern possibly indicates that MG increases belief flexibility and changes the relation of the participants to their symptoms to some extent. As suggested elsewhere (19), the effect of CBT on psychotic disorders is probably of more interest for outcomes such as distress, preoccupation, and conviction related to beliefs than it is for psychotic symptoms as measured by the BPRS. Indeed, through specific training on hypothetical reasoning, MG seems to promote other viewpoints about what happens (as suggested by the observed changes on the PDI-21 and on the MADS), which is considered particularly helpful to patients dealing with stressful positive symptoms (9).

In the present study, as reported in a number of other studies on psychotherapy (36–43), post-intervention effects were sustained at follow-up on the primary outcome. The further improvements observed at follow-up on some outcomes possibly suggests that the effect of the intervention fosters additional changes. One may hypothesize that the first improvements on conviction and the patient’s possible appropriation of the model of reasoning with hypotheses may allow additional experiences in daily life, and then improvements for at least some patients, as suggested by a previous case report (44). Future studies are needed, however, in order to assess the process that may be involved in such supposed effects.

One can also hypothesize that the game could be used as a warm-up program before applying it to an individualized CBT intervention for psychosis. The fidelity checks indicate good fidelity to the intervention in the assessed centers. These results show that an easy-to-use program such as MG may facilitate the dissemination of CBT (specifically hypothetical reasoning training) for psychosis in natural settings. The game may also familiarize health care professionals with the use of CBT techniques for psychosis. This of particularly high interest in consideration of the scarcity of psychotherapeutic treatments offered in clinical settings for people with psychotic disorders (16, 17, 45).

Dropout was relatively low. The game format may increase therapeutic alliance, a possible mediating factor of treatment outcomes in patients with psychosis (46). This aspect was not studied in the present paper, however, and may merit further studies. Dropout at T2 was associated with variables such as a younger age and a higher percentage of participants who dismissed the belief in response to a hypothetical contradiction. These patients possibly perceived less utility in the game than other patients did. The dropout rate associated with younger participants is in accordance with other observations (47) and suggests that the module could be improved to better engage them in the intervention or could be completed with other recovery-oriented interventions and psycho-education (20, 48).

In the absence of an active control group in our study, we cannot exclude the possibility that the observed effects were due to non-specific psychotherapeutic effects such as non-specific attention and interactions with the game leaders and other patients (45, 49). In addition, the absence of audiotaped sessions in some centers to control for fidelity to the intervention by group leaders may have missed poor fidelity in some centers. The lack of precise comparison of the TAU components in the 16 centers may have influenced the results. However, the analyses were controlled for center to correct for a possible cluster effect.

The multicentre design of the study may increase the possible generalization of the results. Furthermore, as reported in other studies (50), the limited number of exclusion criteria could contribute to the external validity of the study. We cannot fully rule out the influence of factors such as comorbid disorders (i.e., substance use disorders) or variations in cognitive deficits on the treatment outcomes.

## Conclusion

The results of this study indicate that the use of MG, a hypothetical reasoning training module, may improve belief flexibility and self-assessment of delusion in patients with psychotic disorders in comparison with not using it. MG appears to be a short and easy-to-use intervention to disseminate CBT for psychosis in routine clinical settings. Further studies with active control group are still needed to improve the understanding of the possible effect of the game.

## Conflict of Interest Statement

Yasser Khazaal and Jerôme Favrod are the authors of the game. The other co-authors declare that the research was conducted in the absence of any commercial or financial relationships that could be construed as a potential conflict of interest.

## Footnotes

^1^http://www.controlled-trials.com/ISRCTN37178153/

^2^http://www.randomization.com

## References

[1] Lieberman JA Stroup TS McEvoy JP Swartz MS Rosenheck RA Perkins DO Effectiveness of antipsychotic drugs in patients with chronic schizophrenia. N Engl J Med (2005) 353(12):1209–23. 10.1056/NEJMoa051688 16172203

[2] Steel C Tarrier N Stahl D Wykes T Cognitive behaviour therapy for psychosis: the impact of therapist training and supervision. Psychother Psychosom (2012) 81(3):194–5 10.1159/000334250 22433800

[3] Tarrier N. Cognitive behaviour therapy for schizophrenia – a review of development, evidence and implementation. Psychother Psychosom (2005) 74(3):136–44. 10.1159/000083998 15832064

[4] Gumley A O’Grady M McNay L Reilly J Power K Norrie J Early intervention for relapse in schizophrenia: results of a 12-month randomized controlled trial of cognitive behavioural therapy. Psychol Med (2003) 33(3):419–31 10.1017/S0033291703007323 12701663

[5] Turkington D Dudley R Warman DM Beck AT. Cognitive-behavioral therapy for schizophrenia: a review. J Psychiatr Pract (2004) 10(1):5–16. 10.1097/00131746-200401000-00002 15334983

[6] Zimmermann G Favrod J Trieu VH Pomini V. The effect of cognitive behavioral treatment on the positive symptoms of schizophrenia spectrum disorders: a meta-analysis. Schizophr Res (2005) 77(1):1–9. 10.1016/j.schres.2005.02.018 16005380

[7] Wykes T Steel C Everitt B Tarrier N. Cognitive behavior therapy for schizophrenia: effect sizes, clinical models, and methodological rigor. Schizophr Bull (2008) 34(3):523–37. 10.1093/schbul/sbm114 17962231 PMC2632426

[8] Rathod S Kingdon D Smith P Turkington D. Insight into schizophrenia: the effects of cognitive behavioural therapy on the components of insight and association with sociodemographics – data on a previously published randomised controlled trial. Schizophr Res (2005) 74(2–3):211–9. 10.1016/j.schres.2004.07.003 15722001

[9] Garety PA Kuipers L Fowler D Chamberlain F Dunn G. Cognitive behavioural therapy for drug-resistant psychosis. Br J Med Psychol (1994) 67(Pt 3):259–71. 10.1111/j.2044-8341.1994.tb01795.x 7803318

[10] Trower P Birchwood M Meaden A Byrne S Nelson A Ross K. Cognitive therapy for command hallucinations: randomised controlled trial. Br J Psychiatry (2004) 184:312–20. 10.1192/bjp.184.4.312 15056575

[11] Wykes T Hayward P Thomas N Green N Surguladze S Fannon D What are the effects of group cognitive behaviour therapy for voices? A randomised control trial. Schizophr Res (2005) 77(2–3):201–10. 10.1016/j.schres.2005.03.013 15885983

[12] Beck AT Rector NA. Cognitive therapy of schizophrenia: a new therapy for the new millennium. Am J Psychother (2000) 54(3):291–300. 11008627 10.1176/appi.psychotherapy.2000.54.3.291

[13] Freeman D Garety PA Fowler D Kuipers E Bebbington PE Dunn G. Why do people with delusions fail to choose more realistic explanations for their experiences? An empirical investigation. J Consult Clin Psychol (2004) 72(4):671–80. 10.1037/0022-006X.72.4.671 15301652

[14] Garety PA Freeman D Jolley S Dunn G Bebbington PE Fowler DG Reasoning, emotions, and delusional conviction in psychosis. J Abnorm Psychol (2005) 114(3):373–84. 10.1037/0021-843X.114.3.373 16117574

[15] Ross K Freeman D Dunn G Garety P. A randomized experimental investigation of reasoning training for people with delusions. Schizophr Bull (2011) 37(2):324–33. 10.1093/schbul/sbn165 19520745 PMC3044626

[16] Kimhy D Tarrier N Essock S Malaspina D Cabannis D Beck A. Cognitive behavioral therapy for psychosis – training practices and dissemination in the United States. Psychosis (2013) 5(3):296–305. 10.1080/17522439.2012.704932 24187582 PMC3811971

[17] Rollinson R Haig C Warner R Garety P Kuipers E Freeman D The application of cognitive-behavioral therapy for psychosis in clinical and research settings. Psychiatr Serv (2007) 58(10):1297–302 10.1176/appi.ps.58.10.1297 17914006

[18] Jones C Cormac I Silveira da Mota Neto JI Campbell C. Cognitive behaviour therapy for schizophrenia. Cochrane Database Syst Rev (2004) (4):CD000524. 10.1002/14651858.CD000524.pub2 15495000

[19] Peters E Landau S McCrone P Cooke M Fisher P Steel C A randomised controlled trial of cognitive behaviour therapy for psychosis in a routine clinical service. Acta Psychiatr Scand (2010) 122(4):302–18. 10.1111/j.1600-0447.2010.01572.x 20491720

[20] Khazaal Y Favrod J Azoulay S Finot SC Bernabotto M Raffard S “Michael’s game,” a card game for the treatment of psychotic symptoms. Patient Educ Couns (2011) 83(2):210–6. 10.1016/j.pec.2010.05.017 20646892

[21] Khazaal Y Favrod J Libbrecht J Finot SC Azoulay S Benzakin L A card game for the treatment of delusional ideas: a naturalistic pilot trial. BMC Psychiatry (2006) 6:48. 10.1186/1471-244X-6-48 17074084 PMC1634845

[22] Leff J Williams G Huckvale MA Arbuthnot M Leff AP. Computer-assisted therapy for medication-resistant auditory hallucinations: proof-of- concept study. Br J Psychiatry (2013) 202:428–33. 10.1192/bjp.bp.112.124883 23429202

[23] Rector NA Beck AT. A clinical review of cognitive therapy for schizophrenia. Curr Psychiatry Rep (2002) 4(4):284–92. 10.1007/s11920-996-0048-5 12126597

[24] Turkington D Kingdon D Weiden PJ Cognitive behavior therapy for schizophrenia. Am J Psychiatry (2006) 163(3):365–73 10.1176/appi.ajp.163.3.365 16513854

[25] Sheehan DV Lecrubier Y Sheehan KH Amorim P Janavs J Weiller E The mini-international neuropsychiatric interview (M.I.N.I.): the development and validation of a structured diagnostic psychiatric interview for DSM-IV and ICD-10. J Clin Psychiatry (1998) 59(Suppl 20):22–33. 9881538

[26] Peters ER Joseph SA Garety PA. Measurement of delusional ideation in the normal population: introducing the PDI (Peters et al. delusions inventory). Schizophr Bull (1999) 25(3):553–76. 10.1093/oxfordjournals.schbul.a033401 10478789

[27] Verdoux H Maurice-Tison S Gay B Van Os J Salamon R Bourgeois ML A survey of delusional ideation in primary-care patients. Psychol Med (1998) 28(1):127–34 10.1017/S0033291797005667 9483688

[28] Pichot P Samuel-Lajeunesse B Lebreaux AM [A new experimental form of BPRS]. Ann Med Psychol (Paris) (1973) 2(2):254–63. 4787811

[29] Shafer A. Meta-analysis of the brief psychiatric rating scale factor structure. Psychol Assess (2005) 17(3):324–35. 10.1037/1040-3590.17.3.324 16262458

[30] Beck AT Baruch E Balter JM Steer RA Warman DM A new instrument for measuring insight: the Beck cognitive insight scale. Schizophr Res (2004) 68(2–3):319–29 10.1016/S0920-9964(03)00189-0 15099613

[31] Favrod J Zimmermann G Raffard S Pomini V Khazaal Y. The Beck cognitive insight scale in outpatients with psychotic disorders: further evidence from a French-speaking sample. Can J Psychiatry (2008) 53(11):783–7. 19087473 10.1177/070674370805301111

[32] Riggs SE Grant PM Perivoliotis D Beck AT. Assessment of cognitive insight: a qualitative review. Schizophr Bull (2012) 38(2):338–50. 10.1093/schbul/sbq085 20693342 PMC3283158

[33] Wessely S Buchanan A Reed A Cutting J Everitt B Garety P Acting on delusions. I: prevalence. Br J Psychiatry (1993) 163:69–76. 10.1192/bjp.163.1.69 8353703

[34] Woods SW. Chlorpromazine equivalent doses for the newer atypical antipsychotics. J Clin Psychiatry (2003) 64(6):663–7. 10.4088/JCP.v64n0607 12823080

[35] Haddock G Devane S Bradshaw T McGovern J Tarrier N Kinderman P An investigation into the psychometric properties of the cognitive therapy scale for psychosis (CTS-Psy). Behav Cogn Psychother (2001) 29(2):221–33.

[36] Steinert C Hofmann M Kruse J Leichsenring F. Relapse rates after psychotherapy for depression – stable long-term effects? A meta-analysis. J Affect Disord (2014) 168C:107–18. 10.1016/j.jad.2014.06.043 25043322

[37] Hsiao FH Jow GM Lai YM Chen YT Wang KC Ng SM The long-term effects of psychotherapy added to pharmacotherapy on morning to evening diurnal cortisol patterns in outpatients with major depression. Psychother Psychosom (2011) 80(3):166–72. 10.1159/000321558 21389753

[38] Cuijpers P Hollon SD van Straten A Bockting C Berking M Andersson G. Does cognitive behaviour therapy have an enduring effect that is superior to keeping patients on continuation pharmacotherapy? A meta-analysis. BMJ Open (2013) 3(4):e002542. 10.1136/bmjopen-2012-002542 23624992 PMC3641456

[39] Ingul JM Aune T Nordahl HM A randomized controlled trial of individual cognitive therapy, group cognitive behaviour therapy and attentional placebo for adolescent social phobia. Psychother Psychosom (2014) 83(1):54–61 10.1159/000354672 24281563

[40] van Ravesteijn H Lucassen P Bor H van Weel C Speckens A. Mindfulness-based cognitive therapy for patients with medically unexplained symptoms: a randomized controlled trial. Psychother Psychosom (2013) 82(5):299–310. 10.1159/000348588 23942259

[41] Khazaal Y Chatton A Prezzemolo R Zebouni F Edel Y Jacquet J Impact of a board-game approach on current smokers: a randomized controlled trial. Subst Abuse Treat Prev Policy (2013) 8:3. 10.1186/1747-597X-8-3 23327643 PMC3564767

[42] Khazaal Y Fresard E Rabia S Chatton A Rothen S Pomini V Cognitive behavioural therapy for weight gain associated with antipsychotic drugs. Schizophr Res (2007) 91(1–3):169–77. 10.1016/j.schres.2006.12.025 17306507

[43] Herbst N Voderholzer U Thiel N Schaub R Knaevelsrud C Stracke S No talking, just writing! Efficacy of an internet-based cognitive behavioral therapy with exposure and response prevention in obsessive compulsive disorder. Psychother Psychosom (2014) 83(3):165–75. 10.1159/000357570 24732962

[44] Khazaal Y Favrod J “Michael’s game” Une approche ludique des troubles psychotiques. J Thér Comportementale Cogn (2012) 22(3):125–9 10.1016/j.jtcc.2012.06.003

[45] Balon R Clinical factor 2012. Psychother Psychosom (2013) 82(4):204–12 10.1159/000351327 23712036

[46] Priebe S Richardson M Cooney M Adedeji O McCabe R. Does the therapeutic relationship predict outcomes of psychiatric treatment in patients with psychosis? A systematic review. Psychother Psychosom (2011) 80(2):70–7. 10.1159/000320976 21196804

[47] Haddock G Lewis S Psychological interventions in early psychosis. Schizophr Bull (2005) 31(3):697–704 10.1093/schbul/sbi029 16006594

[48] Bowersox NW Lai Z Kilbourne AM. Integrated care, recovery-consistent care features, and quality of life for patients with serious mental illness. Psychiatr Serv (2012) 63(11):1142–5. 10.1176/appi.ps.201100505 23117513

[49] Zantvoord JB Diehle J Lindauer RJ. Using neurobiological measures to predict and assess treatment outcome of psychotherapy in posttraumatic stress disorder: systematic review. Psychother Psychosom (2013) 82(3):142–51. 10.1159/000343258 23548778

[50] Kramer U Kolly S Berthoud L Keller S Preisig M Caspar F Effects of motive-oriented therapeutic relationship in a ten-session general psychiatric treatment of borderline personality disorder: a randomized controlled trial. Psychother Psychosom (2014) 83(3):176–86. 10.1159/000358528 24752034

